# Revisiting AFLP fingerprinting for an unbiased assessment of genetic structure and differentiation of taurine and zebu cattle

**DOI:** 10.1186/1471-2156-15-47

**Published:** 2014-04-17

**Authors:** Yuri Tani Utsunomiya, Lorenzo Bomba, Giordana Lucente, Licia Colli, Riccardo Negrini, Johannes Arjen Lenstra, Georg Erhardt, José Fernando Garcia, Paolo Ajmone-Marsan

**Affiliations:** 1 Faculdade de Ciências Agrárias e Veterinárias, UNESP - Univ Estadual Paulista, Jaboticabal, São Paulo 14884-900, Brazil; 2 Institute of Zootechnics, Università Cattolica del Sacro Cuore, Piacenza, Italy; 3 BioDNA Biodiversity and Ancient DNA Research Centre, Università Cattolica del Sacro Cuore, Piacenza, Italy; 4 Faculty of Veterinary Medicine, Utrecht University, Utrecht, Netherlands; 5 Institute of Animal Breeding and Genetics, Justus-Liebig University, Giessen 21b, 35390, Ludwigstrasse, Germany; 6 Faculdade de Medicina Veterinária de Araçatuba, UNESP – Univ Estadual Paulista, Araçatuba, São Paulo 16050-680, Brazil

**Keywords:** Cattle, AFLP, Genetic differentiation, Ascertainment bias

## Abstract

**Background:**

Descendants from the extinct aurochs (*Bos primigenius*), taurine (*Bos taurus*) and zebu cattle (*Bos indicus*) were domesticated 10,000 years ago in Southwestern and Southern Asia, respectively, and colonized the world undergoing complex events of admixture and selection. Molecular data, in particular genome-wide single nucleotide polymorphism (SNP) markers, can complement historic and archaeological records to elucidate these past events. However, SNP ascertainment in cattle has been optimized for taurine breeds, imposing limitations to the study of diversity in zebu cattle. As amplified fragment length polymorphism (AFLP) markers are discovered and genotyped as the samples are assayed, this type of marker is free of ascertainment bias. In order to obtain unbiased assessments of genetic differentiation and structure in taurine and zebu cattle, we analyzed a dataset of 135 AFLP markers in 1,593 samples from 13 zebu and 58 taurine breeds, representing nine continental areas.

**Results:**

We found a geographical pattern of expected heterozygosity in European taurine breeds decreasing with the distance from the domestication centre, arguing against a large-scale introgression from European or African aurochs. Zebu cattle were found to be at least as diverse as taurine cattle. Western African zebu cattle were found to have diverged more from Indian zebu than South American zebu. Model-based clustering and ancestry informative markers analyses suggested that this is due to taurine introgression. Although a large part of South American zebu cattle also descend from taurine cows, we did not detect significant levels of taurine ancestry in these breeds, probably because of systematic backcrossing with zebu bulls. Furthermore, limited zebu introgression was found in Podolian taurine breeds in Italy.

**Conclusions:**

The assessment of cattle diversity reported here contributes an unbiased global view to genetic differentiation and structure of taurine and zebu cattle populations, which is essential for an effective conservation of the bovine genetic resources.

## Background

As a consequence of over 10,000 years of domestication, migrations and natural as well as artificial selection, a wide range of phenotypically distinct cattle populations spread around the world. Several research initiatives have combined molecular marker datasets with historic and archaeological records in order to investigate the origin, history, genetic diversity, and differentiation of cattle populations (see Groeneveld *et al*., 2010 [1] for a review on the topic). The collected evidences suggest that domestic cattle descend from the extinct aurochs (*Bos primigenius*) and are divided into two distinct but interfertile species: the humpless taurine cattle (*Bos taurus*) and the humped indicine or zebu cattle (*Bos indicus*). It is accepted that taurine and zebu cattle have arisen from separate centres of domestication about 8,000 years BC in the Fertile Crescent (modern-day countries of Israel, Jordan, Lebanon, Cyprus and Syria, and parts from Egypt, Turkey, Iraq, Iran and Kuwait) and the Indus valley (current Pakistan), respectively [2,3]. From these regions, cattle have spread throughout Europe, Asia and Africa due to the expansion of agriculture [4,5]. Taurine cattle were imported to the American continent after 1492, mainly from Iberian importations; in the early 20^th^ century, Indian zebu cattle were introduced in Central and South America because of their adaptability to the tropical environment.

Molecular markers have been essential to the investigation of the history and genetic differentiation of domestic cattle. Recent studies applying genome-wide single nucleotide polymorphism (SNP) markers to investigate genetic structure and differentiation in multiple cattle breeds (e.g., [6-9]) resolved hypotheses that were not possible to be tested by using sparse panels of molecular markers. However, markers included in the most widely used SNP panel, the Illumina® BovineSNP50 BeadChip assay (50 k), were discovered in reduced representation libraries from pooled DNA samples of six taurine breeds [10], which leads to biased estimates of genetic structure and differentiation in zebu cattle [7].

As an alternative, amplified fragment length polymorphism (AFLP) markers [11] have been used for almost two decades. Due to their random nature and high reproducibility, they have been enabling ascertainment bias-free analysis of diversity in any species since before the advent of high throughput genotyping and sequencing technologies [12]. AFLP markers are produced by digesting genomic DNA with both a rare cutter and a frequent cutter restriction enzyme, with subsequent ligation of synthetic adapters to the restriction fragments to serve as primer-binding sites, and selective amplification of subsets of the restriction fragments with primers carrying additional nucleotides at their 3’ end [13].

Although AFLP markers are highly informative [13-15] and unbiased, there are few examples of the application of this type of marker in multiple breed, large-scale population differentiation analysis in cattle. Negrini *et al*. [16] used 81 AFLP and 19 microsatellite markers to estimate genetic distances among 51 breeds of cattle, including taurine and zebu cattle, and found that the AFLP panel could differentiate between zebu and taurine cattle better than the panel of microsatellites. Two studies in pigs [17,18] showed the potential of AFLP to survey genetic diversity at the continental scale. Because AFLP polymorphisms are mainly (but not exclusively) based on point mutations, these markers are expected to indicate evolutionary divergence better than microsatellites with variable mutation rates. For instance, a microsatellite-based bovine phylogeny [19] was not in agreement with a phylogeny based on sequence data [20], which was not the case for an AFLP-based phylogeny [21]. Thus, AFLP appears to be a valuable complementary tool for studies of genetic diversity in cattle populations around the world.

Aiming at an unbiased view of genetic structure and differentiation between taurine and zebu cattle breeds from distinct continental areas, we compiled a worldwide multi-breed AFLP dataset. We do not intend to suggest the use of sparse panels of molecular markers over the present portfolio of high-density SNP arrays, or to interrogate their legitimacy for diversity research in cattle. Instead, we intend to propose an unbiased model of cattle differentiation which complements the assessment of genetic distance estimates obtained from molecular markers that are likely to suffer from ascertainment bias.

## Methods

### Sampling and molecular data

A total of 1,593 individuals were genotyped for 135 AFLP markers, representing 13 zebu and 58 taurine breeds. The presence (genotype ‘1’) or absence (genotype ‘0’) of a band was scored considering AFLP as dominant markers, and occasional faint bands were considered as missing data. These samples were obtained from 23 countries from 9 distinct continental areas: Southern Asia (3 zebu breeds), Southwestern Asia (2 taurine breeds), Eastern Europe (3 taurine breeds), Central Europe (24 taurine breeds), Northern Europe (10 taurine breeds), Southern Europe (10 taurine breeds), Western Europe (8 taurine breeds), Western Africa (7 zebu breeds and 1 taurine breed), and South America (3 zebu breeds). This dataset builds on the data reported by Negrini *et al*. [16] by inclusion of samples of 20 additional breeds (Table 1). Individuals or markers presenting 5% or more missing data were excluded from the study. Further details on the AFLP protocol and repeatability of the genotypes obtained can be found in Additional file 1.

**Table 1:** Continental areas, countries and breeds of taurine and zebu cattle sampled

**Continental area**	**Country**	**Breed**	**Zebu**	**Taurine**	**Code**	**n**^**a**^	**n QC**^**b**^
Southern Asia	India	Hariana	x		HAR	4	4
		Tharparkar	x		THA	4	4
	Pakistan	Sahiwal	x		SHA	4	4
Southwestern Asia	Turkey	Anatolian Black		x	ANB	24	23
		Turkish Gray		x	TGS	24	23
Eastern Europe	Hungary	Hungarian Gray		x	HUG	22	19
	Croatia	Istrian		x	ISR	24	23
	Poland	Polish Red		x	POR	23	21
Central Europe	Belgium	Belgian Blue		x	BEB	27	24
	France	Blond d'Aquitaine		x	BLM	20	19
		Bretonne Pie Noir		x	BPN	22	19
		Charolais		x	CHA	22	21
		French Limousine		x	LIM	25	21
		Jersey		x	JER	18	14
		Maine-Anjou		x	MAI	20	18
		Montbéliard		x	MON	22	22
		Normande		x	NOR	23	22
		Parthenaise		x	PAR	15	15
		Salers		x	SAL	20	20
	Switzerland	Brown Swiss		x	SWB	23	20
		Eringer		x	ERI	19	19
		Evoléne		x	EVO	9	8
		Simmentaler		x	SIM	21	19
	Italy	Bruna		x	BRU	33	29
		Frisona		x	FRI	47	44
		Grigio Alpina		x	GAL	21	19
		Italian Limousine		x	LMI	22	19
		Piedmontese*		x	PIM	22(21*)	21
		Pezzata Rossa Italiana		x	PRI	22	22
		Rendena*		x	REN	24(22*)	24
		Valdostana Pezzata Rossa		x	VPR	22	22
	Germany	Original German Black Pied		x	GBP	20	20
	Austria	Pinzgauer		x	PIG	24	22
Northern Europe	England	Aberdeen Angus		x	ABA	20	15
	Norway	Blacksided Trondheim		x	BTR	22	21
		Telemark		x	TEL	22	22
		Vestland Red Polled		x	VPO	22	18
	Denmark	Danish Red		x	DAR	22	21
		Jutland		x	JUT	22	18
	Finland	Eastern Finn Cattle		x	EFC	22	21
		Finnish Ayrshire		x	FAY	22	20
	Iceland	Iceland Cattle		x	ICE	22	22
	Sweden	Swedish Red Polled		x	SRP	22	20
Southern Europe	Italy	Cabannina*		x	CAB	22(20*)	20
		Calvana		x	CAL	40	38
		Chianina*		x	CHI	22(21*)	20
		Cinisara		x	CIN	8	7
		Marchigiana		x	MCG	22	20
		Maremmana		x	MAR	45	45
		Modicana		x	MOD	12	12
		Mucca Pisana		x	MUP	40	39
		Podolica*		x	POD	22(22*)	20
		Romagnola		x	ROM	20	19
Western Europe	Spain	Asturiana de los Valles		x	RAV	20	19
		Betizu		x	BET	20	18
		DiLidia		x	DLD	20	19
		Menorquina		x	MEN	20	19
		Rubia Gallega		x	RUG	20	20
		Sayaguesa		x	SAY	20	19
		Tudanca		x	TUD	20	18
Western Africa	Cameroon	Banyo Gudali	x		CBG	26	18
		Cameronian Red Bororo	x		CRB	25	20
		Cameronian White Fulani	x		CWF	23	23
		Ngaoundere Gudali	x		CNG	25	18
	Guinea-Bissau	Guinean N'Dama		x	GND	20	19
	Nigeria	Red Bororo	x		NRB	25	24
		Sokoto Gudali	x		NSG	25	25
		White Fulani	x		NWF	25	24
South America	Brazil	Guzerat	x		GUZ	32	32
		Nellore	x		NEL	32	21
		Tabapuã	x		TAB	32	32
Total						1,593	1,470

^a^n: Number of samples before quality control.

^b^n QC: Number of samples after quality control.

Quality control was performed by removing samples with 5% or more missing data.

*Breed/number of individuals used to test the repeatability of AFLP fingerprinting (see Additional files 1, 2, 3, 4, 5, 6, 7, 8 and 9).

Underlined breed names correspond to the samples described previously by Negrini *et al*. [16].

### Genetic distances and distance-based clustering

We used *AFLPsurv v1.0*[22] to calculate three different measures of pairwise genetic distances between populations: F_ST_[23], Nei’s D [24] and Reynolds’ distance [25]. We grouped animals according to breed or continental area. The three Southern Asian breeds were excluded in the analyses for individual breeds because of their low sample size (n = 12). We used the *base* package in *R v2.15.0*[26] to perform spectral decompositions on the matrices of pairwise genetic distances between groups in order to construct low-dimensional representations of the genetic relationships among the surveyed populations. The dissimilarities between pairs of groups were captured in *n*-1 dimensional spaces of *n* observations (eigenvectors), where *n* is the number of groups, via classical multi-dimensional scaling (CMDS) [27]. The proportion of genetic variance explained by each eigenvector was calculated by dividing its respective eigenvalue by the sum of all eigenvalues, and expressed as percentages. Additionally, we applied the Neighbor-Net method to the distance matrices by using *SPLITSTREE v4.13.1*[28].

### Expected heterozygosity and ancestry informative markers

With the particular interest of identifying geographical patterns in the extent of genetic diversity in the cattle breeds analyzed, we used *AFLPsurv v1.0*[22] to calculate expected heterozygosities for each continental area under the assumption of Hardy-Weinberg equilibrium. Essentially, the same values were obtained averaging per area over the expected heterozygosities of the separate breeds (data not shown). Additionally, we applied an *ad hoc* statistic to identify taurine and zebu ancestry informative markers (i.e., AFLP markers with large differences in band presence frequencies between taurine and zebu breeds). For each AFLP marker, we computed the band presence frequency across all breeds, and then calculated the mean for the pool of taurine and zebu breeds. We then calculated the difference in band presence frequency as *Δf* = *f*_*taurine*_ − *f*_*zebu*_. Positive and negative values indicate markers that are informative of taurine or zebu ancestry, respectively. We used thresholds of +0.55 and −0.55 to identify taurine and zebu ancestry informative AFLP markers, respectively. Finally, the average of band presence frequency of informative markers was computed for each breed in order to assess the relative level of taurine/zebu introgression across the investigated breeds.

### Model-based clustering

We estimated individual ancestry coefficients as parameters of a statistical model, following the Bayesian approach implemented in *STRUCTURE v2.2*[29]. This is referred as the admixture model adapted for AFLP markers with independent allele frequencies (see [29,30] for details). Briefly, it is assumed that the genomes of the sampled individuals derive from one or more of *K* ancestral populations, and the proportion of the individuals’ ancestry from each one of these populations is estimated via a Markov chain Monte Carlo algorithm. The assumption that the alleles are independent (i.e. linkage equilibrium) is reasonable in the present study, as the AFLP panel used is sparse and the markers are unlikely to be closely located on the genome. We applied this model from *K* = 1 to *K* = 60, and ran 5 replicates of 150,000 iterations for each analysis after a burn-in of 100,000 iterations [31].

We applied two methods to identify the most likely number of ancestral populations underlying the observed data. The first method uses the ∆*K* statistic described by Evanno *et al*. [32], which is based on the rate of change in the log-likelihood of data between successive *K* values. The second method was abstracted from the approaches for model selection reviewed by Johnson & Omland [33], and is based on the concept of relative likelihood. First, the Akaike Information Criterion (*AIC*) is calculated for each model, from *K* = 1 to *K* = 60, as follows: *AIC* = 2*p* − 2 ln(*L*), where *p* and 1n(*L*) are the number of parameters and the log-likelihood of the estimated model, respectively. Next, *AIC* differences are calculated for each model *i* as *Δ*_*i*_ = *AIC*_*i*_ − *AIC*_min_, where *AIC*_min_ corresponds to the lowest *AIC* among all models; and relative likelihoods are computed as $L_{i}=e^{-1/2\Delta_{i}}$. Then, relative likelihood values are normalized across all *K* models to produce Akaike weights $\omega_{i}=L_{i}/\sum_{j=1}^{K}L_{j}$. These can be interpreted as the probability that the respective model is the one that presents the minimum information loss among all competing models, and was used as an alternative approach to estimate the optimal number of *K*.

## Results

### Quality control

After the exclusion of individuals exhibiting 5% or more missing genotypes, 1,470 animals remained from the initial set of 1,593 (see Table 1 for details). From a total of 135 genotyped AFLP loci, 8 were excluded due to missing data (>5%), and the final set of AFLP markers included 127 loci. As most of the analyses reported hereafter assume marker neutrality, the impact of the inclusion of putative markers under selection in all downstream analyses was evaluated. In all cases, the exclusion of candidate outlier markers resulted in no significant difference in the estimates of genetic distances and ancestry coefficients (Additional file 2). Therefore, all subsequent analyses were conducted using the entire set of 127 markers.

### Genetic distance-based clustering

**Figure 1:**
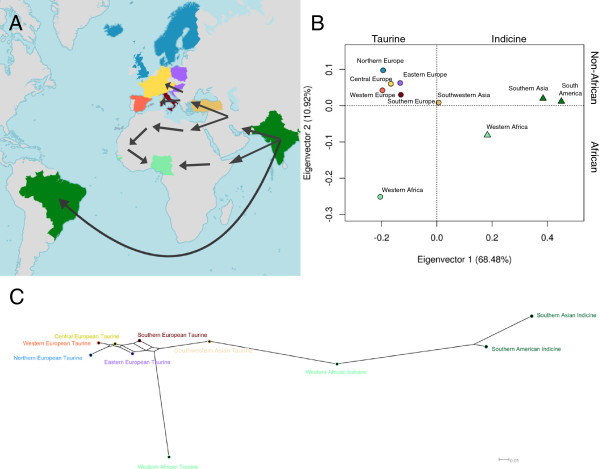
**Reynolds’ distance-based clustering of cattle according to continental areas. A**) Continental areas sampled. Light brown = Southwestern Asia, purple = Eastern Europe, yellow = Central Europe, dark blue = Northern Europe, dark red = Southern Europe, orange = Western Europe, light green = Western Africa, dark green = Southern Asia and South America. Arrows indicate cattle migration routes. **B**) Classical multi-dimensional scaling plot. Circles: taurine cattle; triangles: zebu cattle. Percentages inside brackets correspond to the variance explained by each respective eigenvector. **C**) Neighbor-Net clustering. Nodes represent continental areas and edges are proportional to genetic distances.

Different genetic distances were highly correlated (data not shown) and yielded consistent results (Additional file 3; Additional file 4: Figure S1; Additional file 5: Figure S2; Additional file 6: Figure S3; Additional file 7: Figure S4). We present the results obtained from Reynolds’ distance (Figure 1), which was shown to be insensitive to variation in the number of markers [34].

The Nigerian zebu breeds Sokoto Gudali and White Fulani were the closest related populations (Reynolds’ distance = 0.005). In contrast, in spite of a possible contribution of Spanish ancestry to Brazilian cattle, Brazilian and Spanish breeds are well separated with the largest distance between Nellore and the inbred Betizu (Reynolds’ distance = 0.656).

The first two eigenvectors of the CMDS analysis of continental groups of cattle (Figure 1B) explained together 79.4% of the total genetic variance, and were centered on Southwestern Asian taurine cattle. The first eigenvector corresponds to the difference between taurine and zebu cattle with Southern Asian and South American zebu clustered together, and an intermediate position of Western African zebu cattle. The second eigenvector adds a geographical component correlating with the latitude of the region of origin of cattle populations (Figure 1A-B). The Neighbor-Net clustering method produced results similar to those found in the CMDS analysis (Figure 1C).

### Model-based clustering

**Figure 2:**
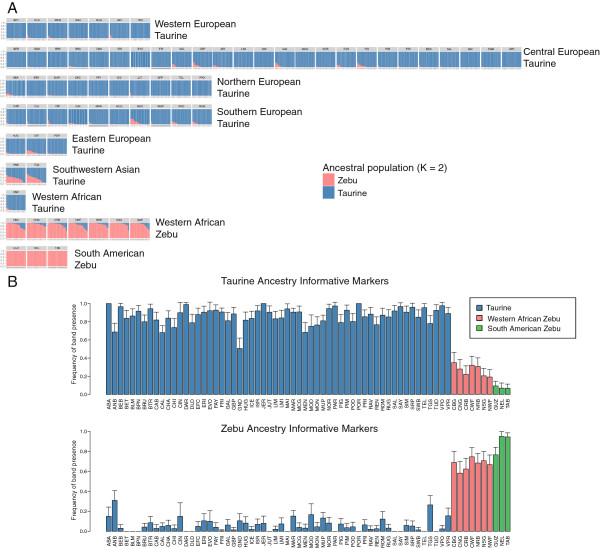
**Admixture analysis of taurine and zebu cattle. A**) Model-based clustering of cattle breeds under the admixture model with independent allele frequencies and 2 assumed ancestral populations (*K*). Each individual is represented by a vertical bar that can be partitioned into colored fragments with length proportional to cluster contribution. **B**) Bar plots of band presence frequencies for the set of taurine (above) and zebu (below) ancestry informative markers. Bar errors represent standard errors. See Table 1 for breed codes.

The log-likelihoods obtained from the admixture model with independent allele frequencies, assuming *K* = 1 to *K* = 60, were compared using ∆*K* and *AIC* weights in order to identify the most likely number of ancestral populations underlying the samples. Both ∆*K* and *AIC* weights selected the model with *K* = 2 as the most likely among all competing models (Additional file 8: Figure S5). Assuming the two inferred clusters approximate the founder *B. taurus* and *B. indicus* populations (Figure 2A), we found variable levels of zebu introgression across taurine cattle breeds from all continental areas, which were especially marked in Southwestern Asian taurines. While South American zebu breeds did not present evident taurine introgression, this was detected in all Western African zebu breeds.

Higher *K* values were not supported by both ∆*K* and *AIC* weights and were not in agreement with genetic distances (data not shown). This indicates that models with K > 2 were susceptible to stochastic errors and represented poorly the underlying ancestry components of our samples. This may be due to model overfitting, by estimation of more parameters than allowed by the observed data. Hence, for our dataset, the model-based clustering analysis was limited to *K* = 2 due to the low number of dominant markers and estimation of unobserved genotypes.

### Ancestry informative markers and expected heterozygosities

We identified 6 taurine and 5 zebu ancestry informative markers via ∆*f*, and calculated the average band presence frequency for these markers across all breeds (Figure 2B). We observed that the taurine markers had in Western African zebus a higher frequency of band presence than in South American zebus, and the opposite was also found for zebu markers.

We found a geographical pattern of decrease in the expected heterozygosity in taurine cattle, declining from Southwestern Asia to Western Europe and Western Africa (Additional file 9: Figure S6). Despite the limited sample size, Southern Asian zebus were estimated to be more diverse than the pools of taurine breeds. The estimate obtained for the closely related South American zebu was slightly lower than in Southwestern Asian taurines, but still higher than in European cattle. Furthermore, Southern Asian and Western African zebus exhibited the highest expected heterozygosity among all continental groups analyzed.

## Discussion

The performance of AFLP technology in cattle was previously assessed and reported to produce genotyping data with an error rate equal to or less than 2% across laboratories [16], which is consistent with the repeatability of the data reported in the present study (Additional file 1). Here, we revisited the use of AFLPs to investigate the relationship among 13 zebu and 53 taurine cattle breeds. As AFLP markers are discovered as samples are genotyped, the assessment of genetic structure and differentiation reported in this article is free of ascertainment bias.

As expected, the largest genetic distances were found between zebu and taurine breeds (Additional file 3). The Bayesian-clustering analysis also highlighted that these populations descend from distinct genetic pools (Figure 2). We found a decrease of the genetic diversity correlating with geographical distance to Southwestern Asia (Additional file 9: Figure S6). This observation is in agreement with the mitochondrial DNA (mtDNA) findings of Troy *et al*. [35], which suggested a Southwestern Asian origin of European cattle with Anatolia or the Fertile Crescent as the most likely centre of taurine cattle domestication. Hence, the loss of diversity with increasing distance from the most plausible domestication centre as observed here is in line with the hypothesis that the ancestral taurine genetic pool was derived from the wild aurochs captured in Southwestern Asia. Apparently, any introgression from European or African aurochs was not at such a large scale that it effectively counteracted the loss of diversity during migration from Southwestern Asia.

Using sequence data of 17 genes, spanning 37 kb, Murray *et al*. [36] found the nucleotide and haplotype diversity in *B. indicus* to be higher than in *B. taurus*. In the present study, we also found that the expected heterozygosity in the South American zebu breeds was higher than in the European taurine breeds. Considering that the South American zebu breeds analyzed here were introduced in the American continent in the early 20^th^ century by import of Indian animals, this finding is also consistent with a separate origin of *B. indicus* in South Asia.

The expected heterozygosity in Southern Asian cattle was estimated to be higher than the closely related South American breeds. Although this finding is consistent with loss of diversity during sampling and importation of animals to South America, Southern Asian cattle were represented by few samples in our dataset, and the assessment of the extent of genetic diversity in this continental group is limited. However, these results support that the *B. indicus* species are at least as diverse as *B. taurus* cattle.

The CMDS and Neighbor-Net analyses showed that zebu cattle from South America are more closely related to Southern Asian cattle than Western African zebu (Figure 1). Furthermore, except for Southern Asian zebus, Western African zebu breeds presented the highest expected heterozygosity among all continental groups. Most likely, this was due to a relatively higher level of admixture [5,37,38].

The closer proximity of Western African zebu to taurine cattle in the CMDS plot and in the Neighbor-Net of Reynolds’ distances also suggests that African zebus are more admixed with taurine cattle than South American zebus (Figure 1). This observation is reinforced by the model-based clustering and the ancestry informative markers analyses, where these African breeds seemed to carry substantial levels of taurine introgression (Figure 2). This may reflect that zebu cattle and taurine-zebu crossbreds in Africa resulted from crosses between taurine dams and zebu sires as shown by their taurine mtDNA haplotypes: import of zebu sires started in the 2^nd^ millennium BC and was stimulated by the Arabian invasions in the 7^th^ century [4,39]. However, it is also plausible that this taurine inheritance played a role in local adaptation. For instance, trypanosomiasis is endemic in the Western Sub-Saharan region, and whereas indigenous taurines are tolerant, zebus may show variable susceptibility.

Similar crossbreeding was carried out in South America. When in the early 20^th^ century the import of large numbers of zebu cattle to Brazil started, the indigenous herds mainly consisted of descendants from the taurine cattle imported since the late 15^th^ century after the discovery of America. The model-based clustering analysis clearly showed a genetic composition of Brazilian zebu close to their Indian ancestors (Figure 2A-B), indicating intensive backcrossing to zebu bulls during several generations. So while mtDNA is a fingerprint of the historical origin of the herd and is probably randomly segregating [40,41], the nuclear genome has been subject to directional selection against taurine haplotypes via backcrossing. Thus, artificial selection may have retained taurine haplotypes only if these were linked to favourable traits (e.g., weight, carcass, etc.). Applying whole genome sequence data or a high density SNP array may be useful to identify taurine haplotypes favoured by selection in these populations.

Ancestry informative markers also detected zebu introgression in the taurine gene pool (Figure 2). The highest level of introgression was found in Southwestern Asia, as previously observed with microsatellites [37]. This event likely contributed to the highest diversity that is observed in this area and, therefore, should not be attributed entirely to the vicinity of Southwestern Asian breeds to the putative *B. taurus* centre of domestication. A low level of admixture was also detected in Southern and Central Italian breeds, the Sicilian Cinisara and Modicana in particular, confirming a previous report [42]. The zebu admixture appears to decrease across the Alps towards Central and Western Europe with few exceptions (e.g., Aberdeen Angus). Interestingly, we confirmed the low level of *B. indicus* introgression in Pinzgauer breed postulated by Caroli *et al*. [43] on the basis of casein haplotype structure in Austria, but did not detect substantial zebu ancestry in the Piedmontese breed as previously suggested [44]. Given the limited number of ancestry informative markers (5 zebu and 6 taurine), these results are only indicative and can be confounded by stochastic variation.

## Conclusions

We used AFLP markers to set an unbiased baseline for multi-breed taurine and zebu cattle genetic structure and divergence. These markers suggested that zebu breeds are at least as diverse as taurine cattle, but further investigation is needed to determine if zebu cattle is more diverse than taurine cattle. We found a gradual loss of diversity in taurine breeds departing from the domestication centre, which is consistent with previous findings. Western African zebu breeds are more genetically distant to Indian zebus than South American zebu cattle by substantial taurine introgression. Although the South American zebus also have maternal taurine introgression, most of the taurine component of the nuclear genome seems to have disappeared through backcrossing. Furthermore, the AFLP data indicated limited zebu introgression in the Italian Podolian breeds.

## Abbreviations

SNP: Single nucleotide polymorphism; 50 k: Illumina® BovineSNP50 BeadChip assay; AFLP: Amplified fragment length polymorphism; CMDS: Classical multi-dimensional scaling; AIC: AKAIKE information criterion; mtDNA: Mitochondrial DNA.

## Competing interests

The authors declare that they have no competing interests.

## Authors’ contributions

PAM and JFG conceived and led the coordination of the study. The European Cattle Genetic Diversity Consortium contributed with data. LC, RN, JAL and GE contributed to the study design. YTU, LB, GL and PAM performed data analyses. YTU drafted the manuscript. PAM, JFG, YTU, LB, JAL, LC, RN and GE interpreted the results and contributed to edit the manuscript. All authors read and approved the final manuscript.

## Supplementary Material

Additional file 1 AFLP protocol and repeatability. Click here for file

Additional file 2 Bias and inflation of non-neutral markers. Click here for file

Additional file 3 **Matrices of pairwise genetic distances between cattle breeds and continental areas.** BIND = *B. indicus*, BTAU = *B. taurus*, SWA = Southwestern Asia, EE = Eastern Europe, CE = Central Europe, NE = Northern Europe, SE = Southern Europe, AF = Africa, AS = Asia, SA = South America. See Table 1 for breed codes. Click here for file

Additional file 4: Figure S1 Classical multi-dimensional scaling analysis between continental areas using three different measures of genetic distance. Percentages inside brackets correspond to the variance explained by the eigenvector. Abbreviations as for Additional file 1. Click here for file

Additional file 5: Figure S2 Classical multi-dimensional scaling analysis between cattle breeds using three different measures of genetic distance. Percentages inside brackets correspond to the variance explained by each respective eigenvector. See Table 1 for breed codes. Click here for file

Additional file 6: Figure S3 Neighbor-net clustering of cattle breeds according to continental area using three different measures of genetic distance. Abbreviations as for Additional file 1. Click here for file

Additional file 7: Figure S4 Neighbor-net clustering of cattle breeds using three different measures of genetic distance. See Table 1 for breed codes. Click here for file

Additional file 8: Figure S5 Model selection for the most probable number of ancestral populations according to two criteria (see Methods). Click here for file

Additional file 9: Figure S6 Bar plot of expected heterozygosities for each continental area. Error bars represent standard errors. Abbreviations as for Additional file 1. Click here for file

## References

[B1] Groeneveld LF Lenstra JA Eding H Toro MA Scherf B Pilling D Negrini R Finlay EK Jianlin H Groeneveld E Weigend S GLOBALDIV Consortium Genetic diversity in farm animals - a review Anim Genet 2010 41 6 31 20500753 10.1111/j.1365-2052.2010.02038.x

[B2] Bruford MW Bradley DG Luikart G Genetic analysis reveals complexity of livestock domestication Nat Rev Genet 2003 4 900 910 14634637 10.1038/nrg1203

[B3] Loftus RT MacHugh DE Bradley DG Sharp PM Cunningham P Evidence for two independent domestications of cattle Proc Natl Acad Sci U S A 1994 91 2757 2761 8146187 10.1073/pnas.91.7.2757 PMC43449

[B4] Ajmone-Marsan P Garcia JF Lenstra JA Globaldiv Consortium On the origin of cattle: how aurochs became cattle and colonized the world Evol Anthropol 2010 19 148 157

[B5] Hanotte O Bradley DG Ochieng JW Verjee Y Hill EW Rege JEO African pastoralism: genetic imprints of origins and migrations Science 2002 296 336 339 11951043 10.1126/science.1069878

[B6] Bovine HapMap C Gibbs RA Taylor JF Van Tassell CP Barendse W Eversole KA Gill CA Green RD Hamernik DL Kappes SM Lien S Matukumalli LK McEwan JC Nazareth LV Schnabel RD Weinstock GM Wheeler DA Ajmone-Marsan P Boettcher PJ Caetano AR Garcia JF Hanotte O Mariani P Skow LC Sonstegard TS Williams JL Diallo B Hailemariam L Martinez ML Morris CA Genome-wide survey of SNP variation uncovers the genetic structure of cattle breeds Science 2009 324 5926 528 532 19390050 10.1126/science.1167936 PMC2735092

[B7] Decker JE Pires JC Conant GC McKay SD Heaton MP Chen K Cooper A Vilkki J Seabury CM Caetano AR Johnson GS Brenneman RA Hanotte O Eggert LS Wiener P Kim JJ Kim KS Sonstegard TS Van Tassell CP Neibergs HL McEwan JC Brauning R Coutinho LL Babar ME Wilson GA McClure MC Rolf MM Kim J Schnabel RD Taylor JF Resolving the evolution of extant and extinct ruminants with high-throughput phylogenomics Proc Natl Acad Sci 2009 106 18644 18649 19846765 10.1073/pnas.0904691106 PMC2765454

[B8] Gautier M Laloë D Moazami-Goudarzi K Insights into the genetic history of French cattle from dense SNP data on 47 worldwide breeds PLoS One 2010 5 e13038 20927341 10.1371/journal.pone.0013038 PMC2948016

[B9] McTavish EJ Decker JE Schnabel RD Taylor JF Hillis DM New World cattle show ancestry from multiple independent domestication events Proc Natl Acad Sci 2013 110 E1398 406 23530234 10.1073/pnas.1303367110 PMC3625352

[B10] Matukumalli LK Lawley CT Schnabel RD Taylor JF Allan MF Heaton MP O'Connell J Moore SS Smith TP Sonstegard TS Van Tassell CP Development and characterization of a high density SNP genotyping assay for cattle PLoS One 2009 4 e5350 19390634 10.1371/journal.pone.0005350 PMC2669730

[B11] Vos P Hogers R Bleeker M Reijans M van de Lee T Hornes M Frijters A Pot J Peleman J Kuiper M AFLP: a new technique for DNA fingerprinting Nucleic Acids Res 1995 23 4407 4414 7501463 10.1093/nar/23.21.4407 PMC307397

[B12] Bensch S Akesson M Ten years of AFLP in ecology and evolution: why so few animals? Mol Ecol 2005 14 2899 2914 16101761 10.1111/j.1365-294X.2005.02655.x

[B13] Ajmone-Marsan P Valentini A Cassandro M Vecchiotti-Antaldi G Bertoni G Kuiper M AFLP markers for DNA fingerprinting in cattle Anim Genet 1997 28 418 426 9589583 10.1111/j.1365-2052.1997.00204.x

[B14] Ajmone-Marsan P Otsen M Valentini A Bertoni G Kuiper MTR Lenstra JA Genetic distances within and across cattle breeds as indicated by biallelic AFLP markers Anim Genet 2002 33 280 286 12139507 10.1046/j.1365-2052.2002.00865.x

[B15] Savelkoul PHM Aarts HJM Dijkshoorn L Duims B De Haas J Otsen M Schouls L Lenstra JA Amplified fragment length polymorphism (AFLP™), the state of an art J Clin Microbiol 1999 37 3083 3091 10488158 10.1128/jcm.37.10.3083-3091.1999 PMC85499

[B16] Negrini R Nijman IJ Milanesi E Moazami-Goudarzi K Williams JL Erhardt G Dunner S Rodellar C Valentini A Bradley DG Ajmone Marsan P Lenstra JA The European Cattle Genetic Diversity Consortium Differentiation of European cattle by AFLP fingerprinting Anim Genet 2007 38 60 66 17257190 10.1111/j.1365-2052.2007.01554.x

[B17] Ollivier L Alderson L Gandini G Foulley JL Haley CS Joosten R Rattink AP Harlizius B Groenen MAM Amigues Y Boscher MY Russel G Law A Davoli R Russo V Matassino D Desautes C Fimland E Bagga M Delgado JV Vega-Pla JL Martinez AM Ramos AM Glodek P Meyer JM Plastow GS Siggens KW Archibald AL Milan D San Cristobal M An assessment of European pig diversity using molecular markers: partitioning of diversity among breeds Conserv Genet 2005 6 729 741

[B18] SanCristobal M Chevalet C Peleman J Heuven H Brugmans B Van Schriek M Joosten R Rattink AP Harlizius B Groenen MA Amigues Y Boscher MY Russell G Law A Davoli R Russo V Dèsautés C Alderson L Fimland E Bagga M Delgado JV Vega-Pla JL Martinez AM Ramos M Glodek P Meyer JN Gandini G Matassino D Siggens K Laval G Genetic diversity in European pigs utilizing amplified fragment length polymorphism markers Anim Genet 2006 37 232 238 16734682 10.1111/j.1365-2052.2006.01440.x

[B19] Ritz LR Glowatzki-Mullis ML MacHugh DE Gaillard C Phylogenetic analysis of the tribe Bovini using microsatellites Anim Genet 2000 31 178 185 10895308 10.1046/j.1365-2052.2000.00621.x

[B20] Hassanin A Ropiquet A Molecular phylogeny of the tribe Bovini (Bovidae, Bovinae) and the taxonomic status of the Kouprey, Bos sauveli Urbain 1937 Mol Phylogenet Evol 2004 33 896 907 15522811 10.1016/j.ympev.2004.08.009

[B21] Buntjer JB Otsen M Nijman IJ Kuiper MT Lenstra JA Phylogeny of bovine species based on AFLP fingerprinting Heredity 2002 88 46 51 11813106 10.1038/sj.hdy.6800007

[B22] Vekemans X Beauwens T Lemaire M Roldan-Ruiz I Data from amplified fragment length polymorphism (AFLP) markers show indication of size homoplasy and of a relationship between degree of homoplasy and fragment size Mol Ecol 2002 11 139 151 11903911 10.1046/j.0962-1083.2001.01415.x

[B23] Wright S The genetical structure of populations Ann Eugen 1951 15 323 354 24540312 10.1111/j.1469-1809.1949.tb02451.x

[B24] Lynch M Milligan BG Analysis of population genetic structure with RAPD markers Mol Ecol 1994 3 91 99 8019690 10.1111/j.1365-294x.1994.tb00109.x

[B25] Reynolds J Weir BS Cockerham CC Estimation of the coancestry coefficient: basis for a short-term genetic distance Genetics 1983 105 767 779 17246175 10.1093/genetics/105.3.767 PMC1202185

[B26] R Development Core Team: R A language and environment for statistical computing 2008 Vienna, Austria: R Foundation for Statistical Computing [http://www.R-project.org]

[B27] Mardia KV Some properties of classical multi-dimensional scaling Commun on Stat-Theory and Methods 1978 A7 1233 1241

[B28] Bryant D Moulton V Neighbor-Net An Agglomerative method for the construction of phylogenetic networks Mol Biol Evol 2004 21 255 265 14660700 10.1093/molbev/msh018

[B29] Pritchard JK Stephens M Donnelly P Inference of population structure using multilocus genotype data Genetics 2000 155 945 959 10835412 10.1093/genetics/155.2.945 PMC1461096

[B30] Falush D Stephens M Pritchard JK Inference of population structure using multilocus genotype data: dominant markers and null alleles Mol Ecol Notes 2007 7 574 578 18784791 10.1111/j.1471-8286.2007.01758.x PMC1974779

[B31] Gilbert KJ Andrew RL Bock DG Franklin MT Kane NC Moore JS Moyers BT Renaut S Rennison DJ Veen T Vines TH Recommendations for utilizing and reporting population genetic analyses: the reproducibility of genetic clustering using the program structure Mol Ecol 2012 21 4925 4930 22998190 10.1111/j.1365-294X.2012.05754.x

[B32] Evanno G Regnaut S Goudet J Detecting the number of clusters of individuals using the software STRUCTURE: a simulation study Mol Ecol 2005 14 2611 2620 15969739 10.1111/j.1365-294X.2005.02553.x

[B33] Johnson JB Omland KS Model selection in ecology and evolution Ecol Evol 2004 19 101 108 10.1016/j.tree.2003.10.013 16701236

[B34] Lenstra JA Groeneveld LF Eding H Kantanen J Williams JL Taberlet P Nicolazzi EL Sölkner J Simianer H Ciani E Garcia JF Bruford MW Ajmone-Marsan P Weigend S Molecular tools and analytical approaches for the characterization of farm animal genetic diversity Anim Genet 2012 43 483 502 22497351 10.1111/j.1365-2052.2011.02309.x

[B35] Troy CS MacHugh DE Bailey JF Magee DA Loftus RT Cunningham P Chamberlain AT Sykes BC Bradley DG Genetic evidence for near-eastern origins of European cattle Nature 2001 41 1088 1091 11323670 10.1038/35074088

[B36] Murray C Huerta-Sanchez E Casey F Bradley DG Cattle demographic history modelled from autosomal sequence variation Philos Trans R Soc Lond B Biol Sci 2010 365 1552 2531 2539 20643743 10.1098/rstb.2010.0103 PMC2935105

[B37] Freeman AR Bradley DG Nagda S Gibson JP Hanotte O Combination of multiple microsatellite data sets to investigate genetic diversity and admixture of domestic cattle Anim Genet 2006 37 1 9 16441289 10.1111/j.1365-2052.2005.01363.x

[B38] Freeman AR Hoggart CJ Hanotte O Bradley DG Assessing the relative ages of admixture in the bovine hybrid zones of Africa and the near east using X chromosome haplotype mosaicism Genetics 2006 173 1503 1510 16582445 10.1534/genetics.105.053280 PMC1526677

[B39] Bradley DG MacHugh DE Cunningham P Loftus RT Mitochondrial diversity and the origins of African and European cattle Proc Natl Acad Sci U S A 1996 93 5131 5135 8643540 10.1073/pnas.93.10.5131 PMC39419

[B40] Meirelles FV Rosa AJM Lobo RB Garcia JM Smith LC Duarte FAM Is the American zebu really *Bos indicus*? Genet Mol Biol 1999 22 543 546

[B41] Paneto JC Ferraz JB Balieiro JC Bittar JF Ferreira MB Leite MB Merighe GK Meirelles FV *Bos indicus* or *Bos taurus* mitochondrial DNA: comparison of productive and reproductive breeding values in a Guzerat dairy herd Genet Mol Res 2008 7 592 602 18752186 10.4238/vol7-3gmr449

[B42] Pieragostini E Scaloni A Rullo R Di Luccia A Identical marker alleles in podolic cattle (*Bos Taurus*) and Indian zebu (*Bos indicus*) Comp Biochem Physiol B Biochem Mol Biol 2000 127 1 9 11126744 10.1016/s0305-0491(00)00218-2

[B43] Caroli A Rizzi R Lühken G Erhardt G Short communication: milk protein genetic variation and casein haplotype structure in the original pinzgauer cattle J Dairy Sci 2009 93 1260 1265 20172246 10.3168/jds.2009-2521

[B44] Merlin P Di Stasio L Study on milk proteins loci in some decreasing Italian cattle breeds Ann Genet Sel Anim 1982 14 17 28 22896222 10.1186/1297-9686-14-1-17 PMC2734723

