# Supplementary material for: Revisiting AFLP fingerprinting for an unbiased assessment of genetic structure and differentiation of taurine and zebu cattle
Source: BMC Genet. 2014 Apr 17;15:47. doi: 10.1186/1471-2156-15-47 (PMC4021504; doi:10.1186/1471-2156-15-47)
Supplement: Additional file 1 — AFLP protocol and repeatability. [file 1471-2156-15-47-S1.pdf]

File S1 - AFLP protocol and repeatability

Sample collection and ethical statement

Blood samples were collected in the course of the EU-funded project “RESGEN: Towards a strategy for the conservation of the genetic diversity of European cattle”. Experimental procedures were approved by the EU Commission, in accordance with the EU Directive 86/609, Resgen contract n. CT98-118, within the FP5-Framework Programme 5. The permission to carry out the sampling at each farm was obtained directly from the owners. Blood samples have been collected by veterinarians, complying with relevant national and international regulations and animal welfare requirements.

DNA extraction

Total DNA was extracted from 200 ul of whole blood using the Genelute Mammalian Genomic DNA Kit (Sigma) using the manufacturer’s instructions.

AFLP protocol

We applied the following highly standardized protocol to produce *Eco*RI/*Taq*I AFLP markers:

Step 1. *Taq*I restriction: 100 ng of DNA was incubated for 1 h at 65°C with 5 U of *Taq*I restriction enzyme (New England BiolabsInc, Ipswich, MA) in a volume of 25 ul containing: 10 mMTris-HAc (pH 7.5), 10 mMMgAc, 50 mMKAc, 5 mMdithiothreitol (DTT) and 50 ng/ul bovine serum albumin (BSA).

Step 2. *Eco*RI restriction:15 ul of a solution having the same DTT, BSA and ion composition described above and containing 5 U of *Eco*RI restriction enzyme (New England BiolabsInc, Ipswich, MA) was added. The resulting 40 ul was incubated at 37°C for 1 h.

Step 3. Ligation:the ligation of adapters was performed by adding 10 ul of a solution containing 5 pmol*Eco*RI adapters, 50 pmol*Taq*I adapters, 1 U of T4 DNA ligase (New England BiolabsInc, Ipswich, MA) and 1 mM ATP, in the same salt, DTT and BSA concentration described above; the final 50 ul were then incubated at 37°C for 3 h.

Step 4. Template DNA dilution: Template DNA was diluted 1:10 before further use.

Step 5. Pre-amplification:5 ul of diluted template DNA was added to 45 ul of the following PCR reaction mix: 1x reaction buffer, 1.5 mM $MgCl_2$, 0.2 mMdNTPs, 1 U of Applied Biosystems® AmpliTaq® DNA Polymerase (Life Technologies Ltd, Paisley, UK) and 75 ng each of *Eco*RIE01 and *Taq*IT01 primers carrying one selective nucleotide (**Table S1.1**). Pre-amplification thermal profile consisted on 30 cycles of 30 s at 94°C, 1 min at 56° and 1 min at 72°C, with a 10 min at 72°C of final extension to allow the completion of partial amplifications fragments.

Step 6. Pre-amplified template dilution: the pre-amplified template was diluted 1:20 and processed further.

Step 7. Selective amplification: starting from 5 ul of diluted pre-amplified template, selective amplification was carried out in a final volume of 20 ul by adding PCR reaction mix containing 1x reaction buffer, 1.5 mM $MgCl_2$, 0.2 mMdNTPs, 0.4 U of Applied Biosystems® AmpliTaq® DNA Polymerase (Life Technologies Ltd, Paisley, UK), 5 ng^{33}P terminally labeled *Eco*RI primer and 30 ngunlabelled*Taq*I primer, both carrying three selective nucleotides each (**Table S1.1**). AFLP fingerprinting was carried out using the following three primer combinations: E35-T32, E39-T33 and E45-T32. To ensure high-stringency amplification, we adopted a thermal profile which includeda touchdown PCR strategy:

Cycle 1: 30 s at 94°C , 30 s at 65°C, 1 min at 72°C.
Cycles 2 to 14: in each cycle, the annealing temperature was reduced by 0.7°C down to 56°C.
Cycles 15 to 36: 30 s at 94°C , 30 s at 56°C, 1 min at 72°C.

Table S1.1. Sequences of the adapters and primers used in AFLP analysis. Selective nucleotides are underlined.

*Eco*RI adapters	*Eco* top strand	CTCGTAGACTGCGTACC
	Eco bottom strand	AATTGGTACGCAGTCTAC
*Eco*RI primers	E01 (pre-amplification)	GAC TGC GTA CCA ATT CA
	E35	GAC TGC GTA CCA ATT CAC A
	E39	GAC TGC GTA CCA ATT CAG A
	E45	GAC TGC GTA CCA ATT CAT G
*Taq*I adapters	*Taq* top strand	GACGATGAGTCCTGAC
	Taq bottom strand	CGGTCAGGACTCAT
*Taq*I primers	T01 (pre-amplification)	GAT GAG TCC TGA CCG AA
	T32	GAT GAG TCC TGA CCG AAA C
	T33	GAT GAG TCC TGA CCG AAA G

Detection and scoring of AFLP markers. ^{33}P terminally labeled amplification products were mixed with an equal amount of formamide loading buffer, denatured and separated on a 40 cm 4.5% polyacrylamide sequencing gel in TBE buffer (100 mMTris-HCl, 100 mM Boric acid, 2 mM EDTA) at a constant wattage of 56 W for 2 hours. The gel was then fixed for 20 min in 10% acetic acid, rinsed with deionized water, dried and autoradiographed on AmershamHyperfilm MP for 48 h (Amersham International, Little Chalfont, UK). AFLP markers were scored visually from autoradiographs in a size range from 50 to 600 bp.

Repeatability. In order to guarantee AFLP profile consistency, the genotyping of 106 total individuals belonging to 5 breeds was carried out starting from different aliquots of DNA in two different labs: at the Laboratory of Animal Genetics of the Università Cattolica del Sacro Cuore, following the same protocol described above, and at KeyGene (http://www.keygene.com/) following their patented protocol. The repeatability of genotypes was evaluated by comparing the individual profiles; the error rate associated with the final dataset was estimated over a matrix of 105 individuals and 127 markers. The replicated individual genotypes were compared to calculate the frequency of mismatches. The results were then plotted as frequency of identity between genotypes (1 –frequency of mismatches) with mean and median values of 0.95 (±0.04 s.d.) and 0.97, respectively. Since the resulting distribution was negatively skewed (**Figure S1.1**), the median value was assumed as a descriptive statistic.

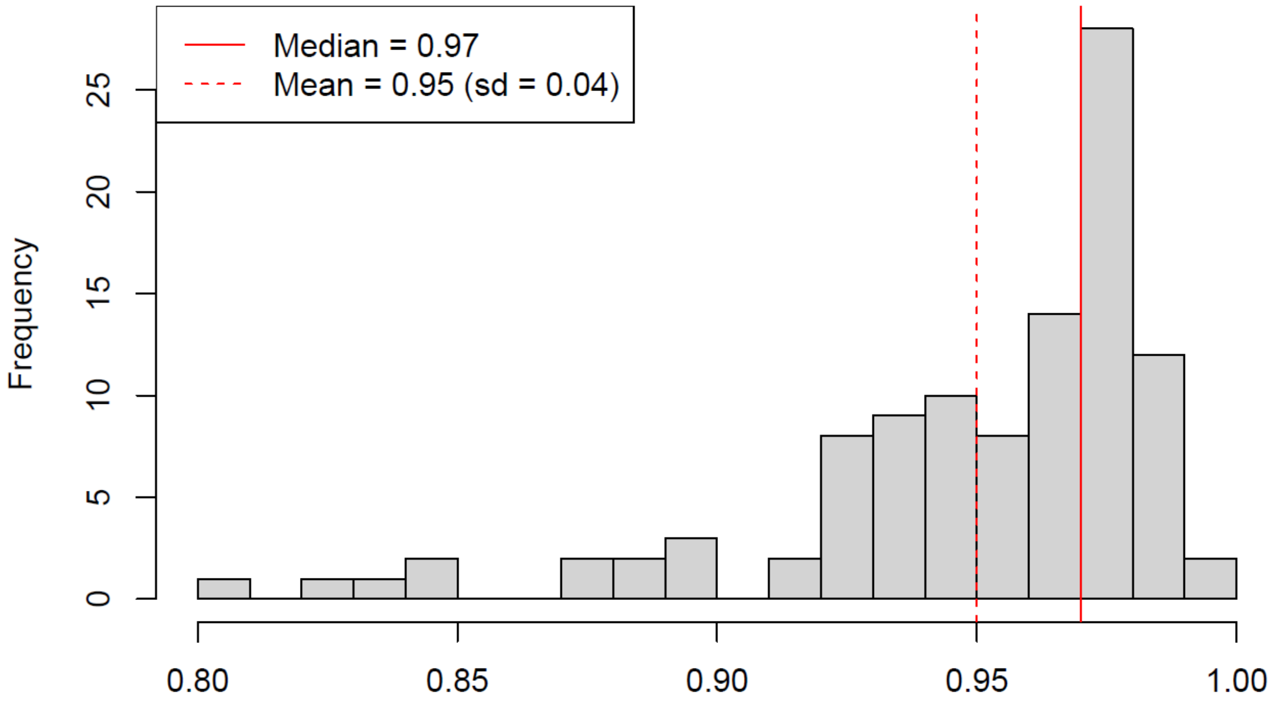

Figure S1.1. Bar plot showing the frequency of identity values calculated between pairs of replicated individual genotypes. The solid red line indicates the mean value of 0.97.

To further test the consistency of the data generated at the different laboratories, Nei, Fst and Reynolds' genetic distances were calculated between replicated breeds. All distances values between replicated breeds were zero (**Table S1.2**).

Table S1.2. Nei, Fst and Reynolds' genetic distances calculated over the dataset of the replicated breeds.

Genetic distances calculated between repeated breeds (PC=Piacenza vs. KEY=Keygene)										
Nei's	genetic	distance	after	Lynch	and	Milligan	-	1994		
	CHI_KEY	PIM_KEY	POD_KEY	CAB_KEY	REN_KEY	CHI_PC	PIM_PC	POD_PC	CAB_PC	REN_PC
CHI_KEY	0.0000	0.0285	0.0238	0.0408	0.0410	0.0000	0.0302	0.0257	0.0390	0.0377
PIM_KEY	0.0285	0.0000	0.0078	0.0079	0.0139	0.0297	0.0000	0.0141	0.0120	0.0154
POD_KEY	0.0238	0.0078	0.0000	0.0127	0.0198	0.0242	0.0131	0.0000	0.0152	0.0167
CAB_KEY	0.0408	0.0079	0.0127	0.0000	0.0130	0.0415	0.0116	0.0209	0.0000	0.0175
REN_KEY	0.0410	0.0139	0.0198	0.0130	0.0000	0.0350	0.0080	0.0194	0.0133	0.0000
CHI_PC	0.0000	0.0297	0.0242	0.0415	0.0350	0.0000	0.0251	0.0248	0.0362	0.0329
PIM_PC	0.0302	0.0000	0.0131	0.0116	0.0080	0.0251	0.0000	0.0175	0.0117	0.0117
POD_PC	0.0257	0.0141	0.0000	0.0209	0.0194	0.0248	0.0175	0.0000	0.0121	0.0125
CAB_PC	0.0390	0.0120	0.0152	0.0000	0.0133	0.0362	0.0117	0.0121	0.0000	0.0107
REN_PC	0.0377	0.0154	0.0167	0.0175	0.0000	0.0329	0.0117	0.0125	0.0107	0.0000
Pairwise	Fst	between	populations							
	CHI_KEY	PIM_KEY	POD_KEY	CAB_KEY	REN_KEY	CHI_PC	PIM_PC	POD_PC	CAB_PC	REN_PC
CHI_KEY	0.0000	0.1091	0.0805	0.1427	0.1469	0.0000	0.1106	0.0837	0.1287	0.1312
PIM_KEY	0.1091	0.0000	0.0282	0.0290	0.0544	0.1124	0.0000	0.0471	0.0421	0.0559
POD_KEY	0.0805	0.0282	0.0000	0.0474	0.0738	0.0850	0.0501	0.0000	0.0513	0.0585
CAB_KEY	0.1427	0.0290	0.0474	0.0000	0.0546	0.1480	0.0494	0.0723	0.0000	0.0680
REN_KEY	0.1469	0.0544	0.0738	0.0546	0.0000	0.1320	0.0366	0.0699	0.0531	0.0000
CHI_PC	0.0000	0.1124	0.0850	0.1480	0.1320	0.0000	0.0925	0.0806	0.1187	0.1160
PIM_PC	0.1106	0.0000	0.0501	0.0494	0.0366	0.0925	0.0000	0.0591	0.0407	0.0436
POD_PC	0.0837	0.0471	0.0000	0.0723	0.0699	0.0806	0.0591	0.0000	0.0414	0.0462
CAB_PC	0.1287	0.0421	0.0513	0.0000	0.0531	0.1187	0.0407	0.0414	0.0000	0.0412
REN_PC	0.1312	0.0559	0.0585	0.0680	0.0000	0.1160	0.0436	0.0462	0.0412	0.0000
Reynolds	et	al.	genetic	distance	between	populations				
	CHI_KEY	PIM_KEY	POD_KEY	CAB_KEY	REN_KEY	CHI_PC	PIM_PC	POD_PC	CAB_PC	REN_PC
CHI_KEY	0.0000	0.1156	0.0840	0.1540	0.1589	0.0000	0.1172	0.0875	0.1377	0.1406
PIM_KEY	0.1156	0.0000	0.0286	0.0295	0.0559	0.1193	0.0000	0.0482	0.0430	0.0576
POD_KEY	0.0840	0.0286	0.0000	0.0486	0.0766	0.0888	0.0514	0.0000	0.0526	0.0603
CAB_KEY	0.1540	0.0295	0.0486	0.0000	0.0562	0.1602	0.0507	0.0750	0.0000	0.0704
REN_KEY	0.1589	0.0559	0.0766	0.0562	0.0000	0.1416	0.0373	0.0725	0.0546	0.0000
CHI_PC	0.0000	0.1193	0.0888	0.1602	0.1416	0.0000	0.0970	0.0841	0.1264	0.1233
PIM_PC	0.1172	0.0000	0.0514	0.0507	0.0373	0.0970	0.0000	0.0609	0.0415	0.0446
POD_PC	0.0875	0.0482	0.0000	0.0750	0.0725	0.0841	0.0609	0.0000	0.0423	0.0473
CAB_PC	0.1377	0.0430	0.0526	0.0000	0.0546	0.1264	0.0415	0.0423	0.0000	0.0421
REN_PC	0.1406	0.0576	0.0603	0.0704	0.0000	0.1233	0.0446	0.0473	0.0421	0.0000
