# Supplementary material for: Revisiting AFLP fingerprinting for an unbiased assessment of genetic structure and differentiation of taurine and zebu cattle
Source: BMC Genet. 2014 Apr 17;15:47. doi: 10.1186/1471-2156-15-47 (PMC4021504; doi:10.1186/1471-2156-15-47)
Supplement: Additional file 3 — Matrices of pairwise genetic distances between cattle breeds and continental areas. BIND = B. indicus, BTAU = B. taurus, SWA = Southwestern Asia, EE = Eastern Europe, CE = Central Europe, NE = Northern Europe, SE = Southern Europe, AF = Africa, AS = Asia, SA = South America. See Table 1 for breed codes. [file 1471-2156-15-47-S3.pdf]

File S3 - Bias and inflation of non-neutral markers

Hardy-Weinberg equilibrium and neutrality are assumptions shared by all measures of genetic distance and structure computed in the present report. In order to evaluate the impact of ignoring departures from these theoretical models, we calculated Reynolds' distance, F_{ST}, Nei's D and ancestry coefficients using two sets of markers: all 127 markers passing quality control (M_{all}) and a reduced panel excluding candidate outlier markers ($M_{neutral}$). Then, we assessed the bias and inflation of the estimates obtained with M_{all} relative to $M_{neutral}$.

Detection of candidate markers under selection

We used the MCHEZA program (Antao et al., 2008; Antao & Beaumont, 2011) to identify putative AFLP markers under selection. Briefly, MCHEZA builds on the methods implemented in DFDIST (Beaumont and Nichols, 1996; Perez-Figueroa et al, 2010), which relies on coalescent simulations to generate distributions of F_{ST} under neutral expectations in a drift model. Markers are declared non-neutral when F_{ST} values are excessively high or low compared to the null simulated distribution.

A limitation of the method is that it cannot reliably identify outlier loci when the populations being compared present an average F_{ST} larger than 0.20 (Pérez-Figueroa et al., 2010). As the average F_{ST} between taurine and zebu breeds was 0.28 in the present study, we analyzed the two species separately. Average F_{ST} among taurine and zebu breeds were 0.12 and 0.11, respectively. The parameter settings used for each analysis can be found in **Table S3.1** (for parameter descriptions, see Antao et al., 2008; Antao & Beaumont, 2011).

Table S3.1 MCHEZA parameter settings used for the detection of AFLP markers under selection.

Parameter	Zebu	Taurine
Simulations to estimate neutral F_{ST}	100,000	100,000
Simulations for neutral distribution	100,000	100,000
Confidence interval	0.99	0.99
False discovery rate	0.01	0.01
Expected total populations	10	58
Subsample size	23	20
Theta[a]	0.1	0.1
Prior distribution for allele frequencies	Beta(0.25,0.25)	Beta(0.25,0.25)
Critical allele frequency	0.99	0.99

[a]Twice the mutation rate (per site per generation) times the number of heritable units in the population

Measure of bias

Let θ denote the set of the true values for any given parameter reported here (Reynolds' distance, F_{ST}, Nei's D or ancestry coefficients), and $\hat{\theta}$ its estimator. The bias of the estimator is defined as $Bias(\hat{\theta}) = E[\hat{\theta} - \theta]$. As quantity θ is unknown, we considered the estimates obtained with $M_{neutral}$ as surrogates for the true values, and $\hat{\theta}$ and M_{all} were used interchangeably. Therefore, the estimation bias introduced by ignoring departures from neutrality was measured as the realized average difference between M_{all} and $M_{neutral}$.

Measure of inflation

One may expect that the inclusion of non-neutral markers may bias the estimates upwards, i.e., may cause overestimation of the genetic parameters. This inflation can be represented by the slope of a regression of M_{all} on $M_{neutral}$, and regression coefficients close to 1 are evidence of no inflation.

Impact of non-neutral markers

A total of 4 and 3 candidate outlier loci under positive selection were identified in the taurine and zebu datasets, respectively, and a single putative marker under balancing selection was detected in the zebu dataset. Therefore, a total of 120 markers remained in the $M_{neutral}$ panel.

Figure S3.1 shows regression scatterplots and **Table S3.2** presents the estimated bias and inflation for all computed statistics. Estimated bias was close to 0 and inflation was close to 1 in all cases. Squared correlations between M_{all} and $M_{neutral}$ were greater than 0.995. Differences obtained between the panels were negligible and could not be discriminated from stochastic variation due to marker sampling. Therefore, we found no evidence supporting that the inclusion of putative markers under selection may have biased the analyses assuming neutrality in the present report.

Table S3.2 Bias and inflation of estimates of genetic distance and structure in scenarios violating the assumption of neutral markers.

Statistic	Bias	Inflation
Reynolds' distance	4×10^{-3}	0.999
FST	4×10^{-3}	0.996
Nei's D	1×10^{-3}	0.983
Ancestry coefficient	1×10^{-4}	0.995

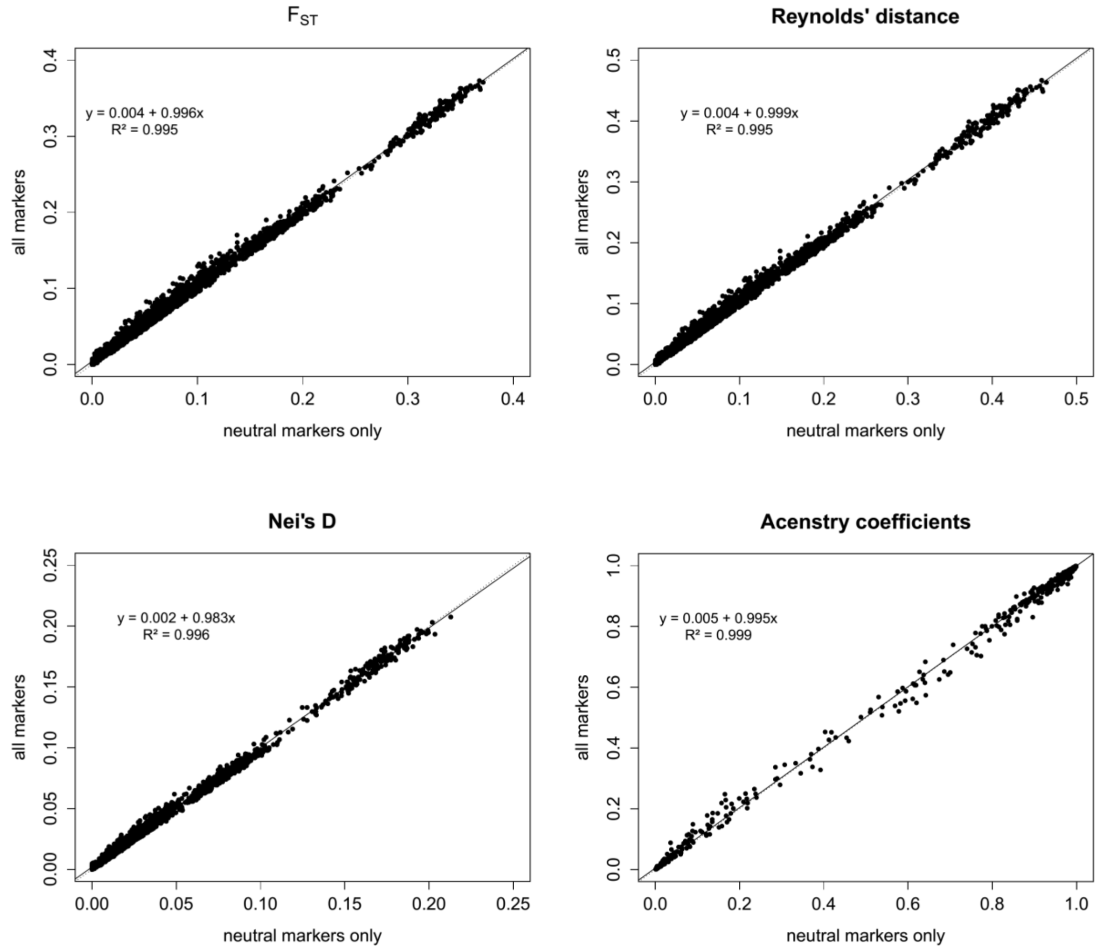

Figure S3.1. Regression scatterplots for measures of genetic distance and structure in a scenario violating (y axis) and in a scenario accounting for (x axis) the assumption of neutral markers. Solid lines represent regression lines resulting from the estimated intercept and slope (indicated in the equations), whereas dotted lines represent a theoretical regression with an intercept of 0 and a slope of 1.

References

1. Antao T, Beaumont MA: **Mcheza: a workbench to detect selection using dominant markers**. *Bioinformatics*. 2011, **27**:1717-1718.
2. Antao T, Lopes A, Lopes RJ, Beja-Pereira A, Luikart G: **LOSITAN: a workbench to detect molecular adaptation based on a Fst-outlier method**. *BMC Bioinformatics*. 2008, **9**:323.
3. Beaumont MA, Nichols RA: **Evaluating loci for use in the genetic analysis of population structure**. *Proceedings of the Royal Society B*. 1996, **263**:1619-1626.
4. Pérez-Figueroa A, García-Pereira MJ, Saura M, Rolán-Alvarez E, Caballero A: **Comparing three different methods to detect selective loci using dominant markers**. *Journal of Evolutionary Biology*. 2010, **23**:2267-2276.
