# Supplementary figures and images for: Revisiting AFLP fingerprinting for an unbiased assessment of genetic structure and differentiation of taurine and zebu cattle
Source: BMC Genet. 2014 Apr 17;15:47. doi: 10.1186/1471-2156-15-47 (PMC4021504; doi:10.1186/1471-2156-15-47)

**Reynolds' distance**

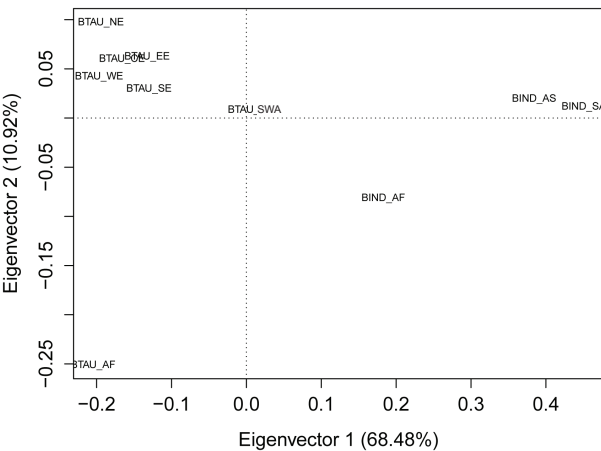

**F<sub>ST</sub>**

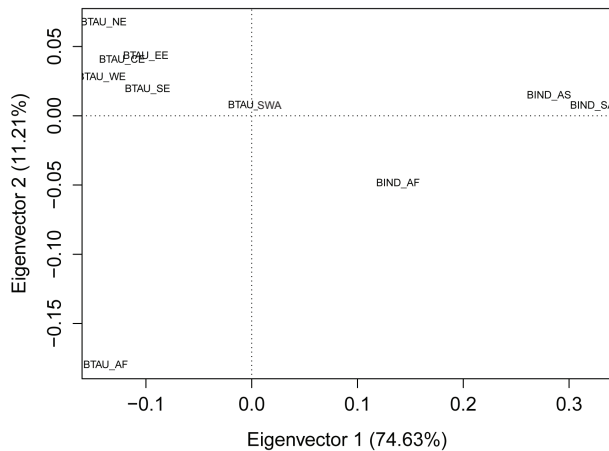

**Nei's D**

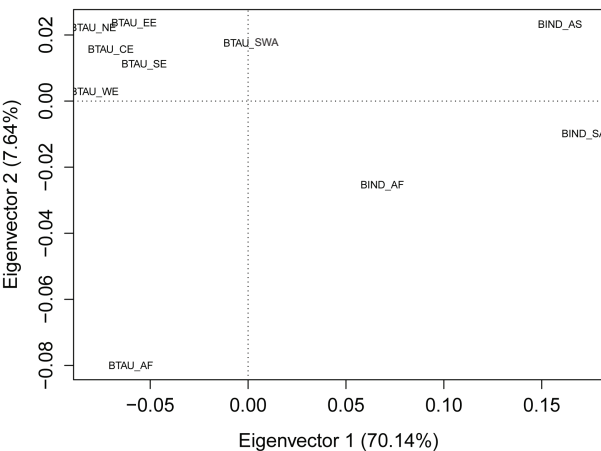

Supplement: Additional file 4: Figure S1 — Classical multi-dimensional scaling analysis between continental areas using three different measures of genetic distance. Percentages inside brackets correspond to the variance explained by the eigenvector. Abbreviations as for Additional file 1. [file 1471-2156-15-47-S4.pdf]

**Reynolds' distance**

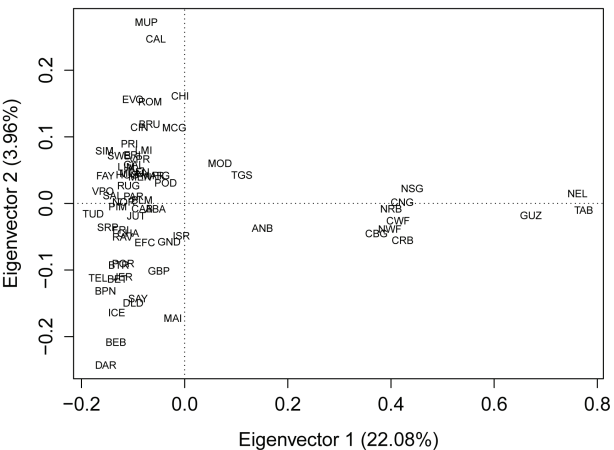

**F<sub>ST</sub>**

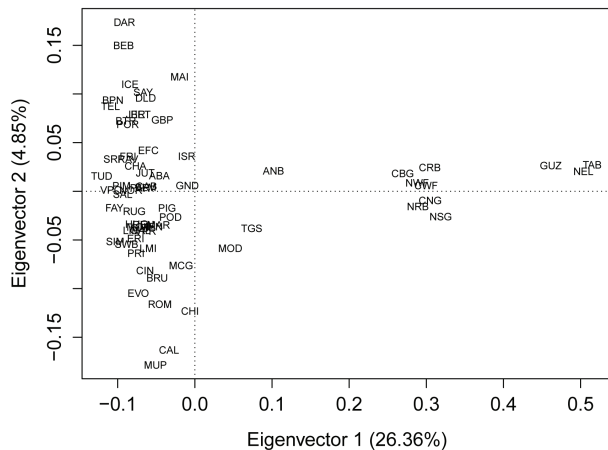

**Nei's D**

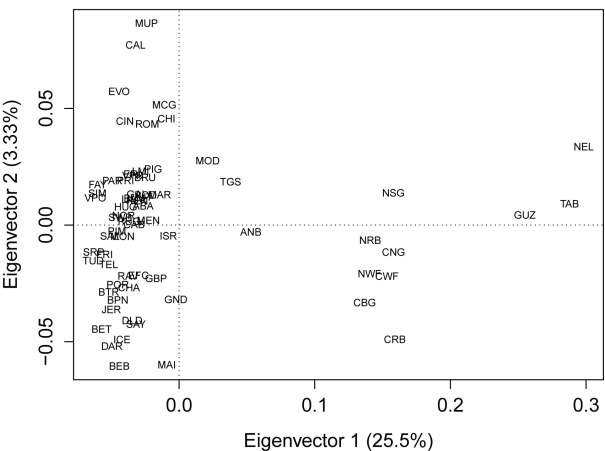

Supplement: Additional file 5: Figure S2 — Classical multi-dimensional scaling analysis between cattle breeds using three different measures of genetic distance. Percentages inside brackets correspond to the variance explained by each respective eigenvector. See Table 1 for breed codes. [file 1471-2156-15-47-S5.pdf]

# Reynolds' distance

0.01

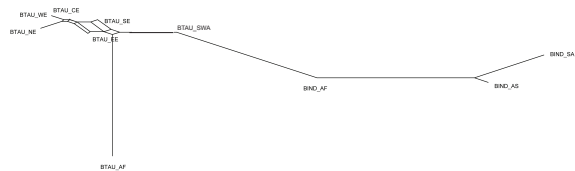

0.01

# F<sub>ST</sub>

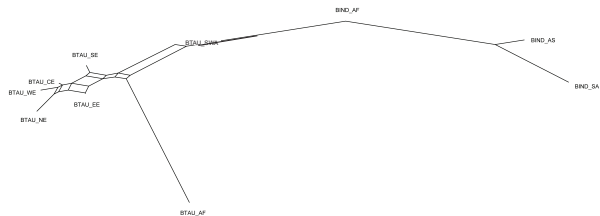

0.01

# Nei's D

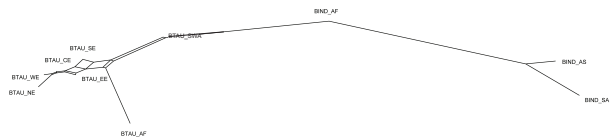

Supplement: Additional file 6: Figure S3 — Neighbor-net clustering of cattle breeds according to continental area using three different measures of genetic distance. Abbreviations as for Additional file 1. [file 1471-2156-15-47-S6.pdf]

Evanno *et al.* (2005)

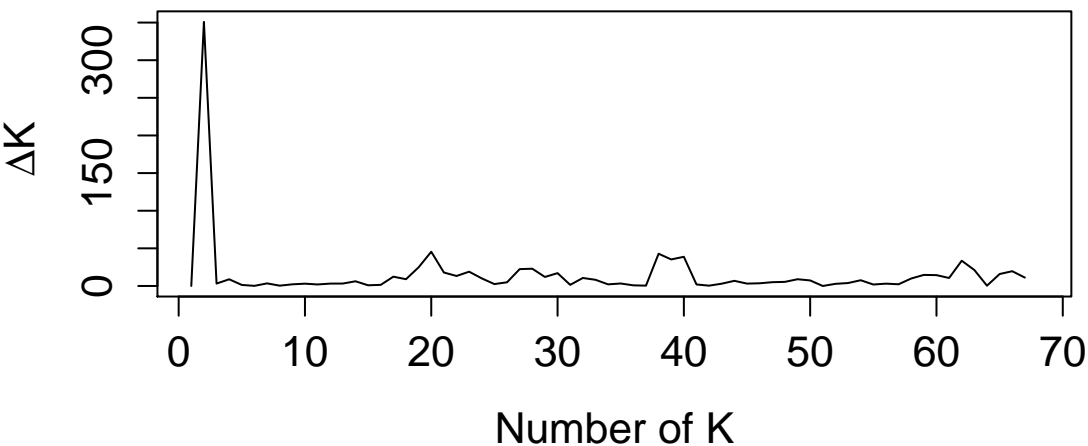

AIC weights

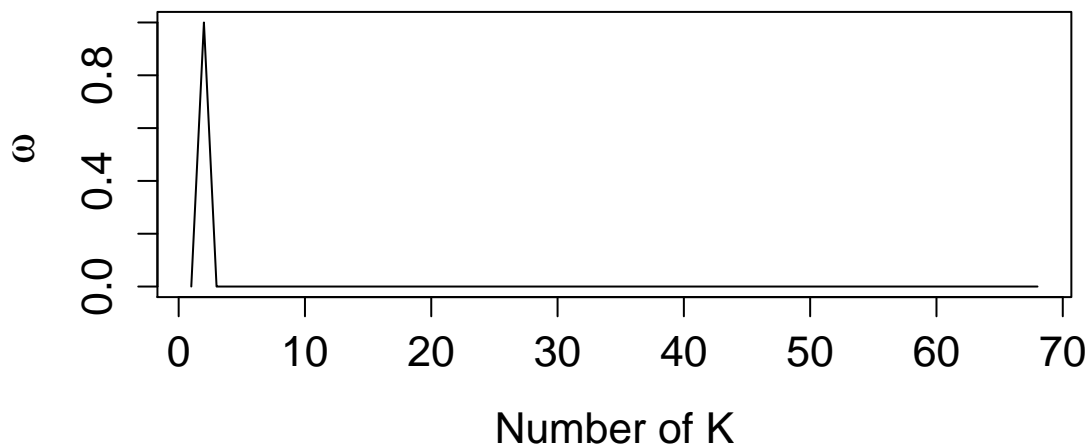

Supplement: Additional file 8: Figure S5 — Model selection for the most probable number of ancestral populations according to two criteria (see Methods). [file 1471-2156-15-47-S8.pdf]

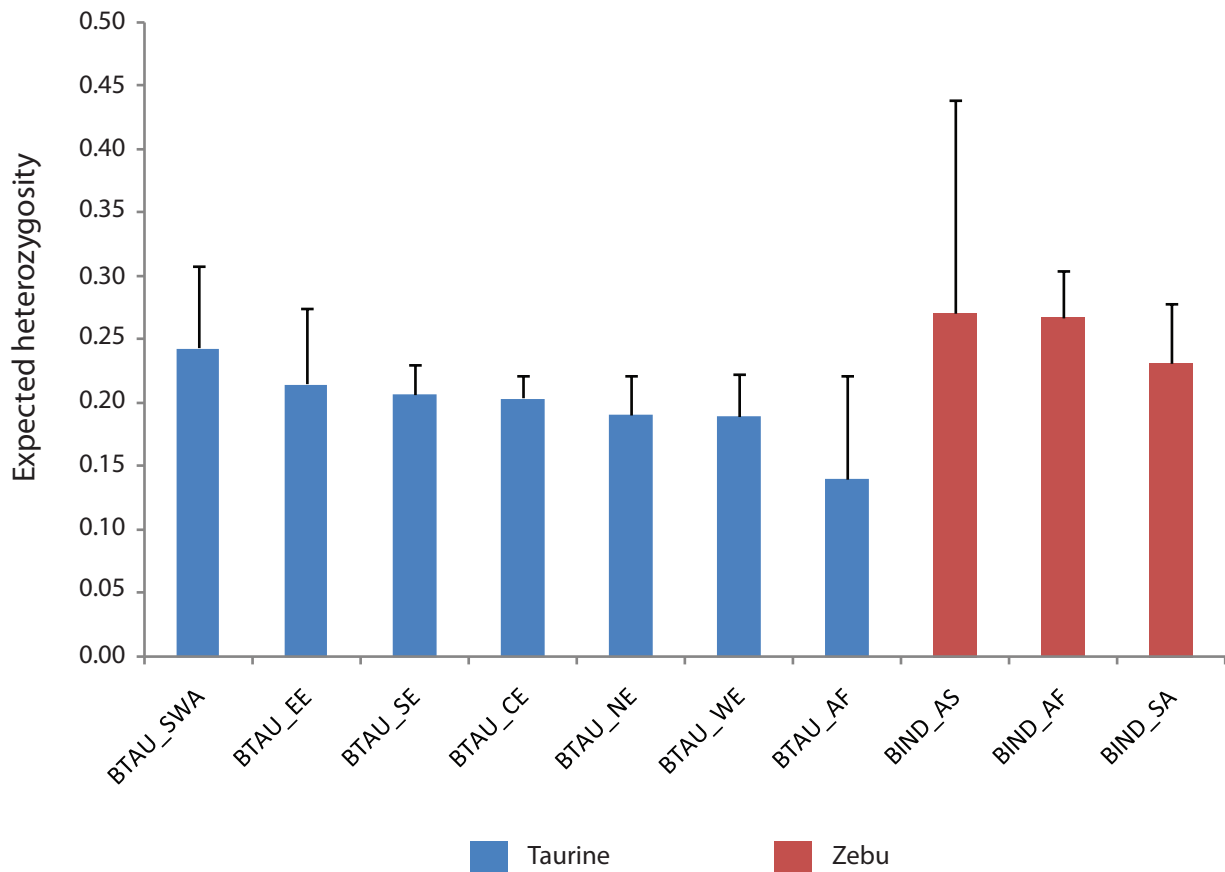

Supplement: Additional file 9: Figure S6 — Bar plot of expected heterozygosities for each continental area. Error bars represent standard errors. Abbreviations as for Additional file 1. [file 1471-2156-15-47-S9.pdf]
