# Supplementary material for: Revisiting AFLP fingerprinting for an unbiased assessment of genetic structure and differentiation of taurine and zebu cattle
Source: BMC Genet. 2014 Apr 17;15:47. doi: 10.1186/1471-2156-15-47 (PMC4021504; doi:10.1186/1471-2156-15-47)
Supplement: Additional file 7: Figure S4 — Neighbor-net clustering of cattle breeds using three different measures of genetic distance. See Table 1 for breed codes. [file 1471-2156-15-47-S7.pdf]

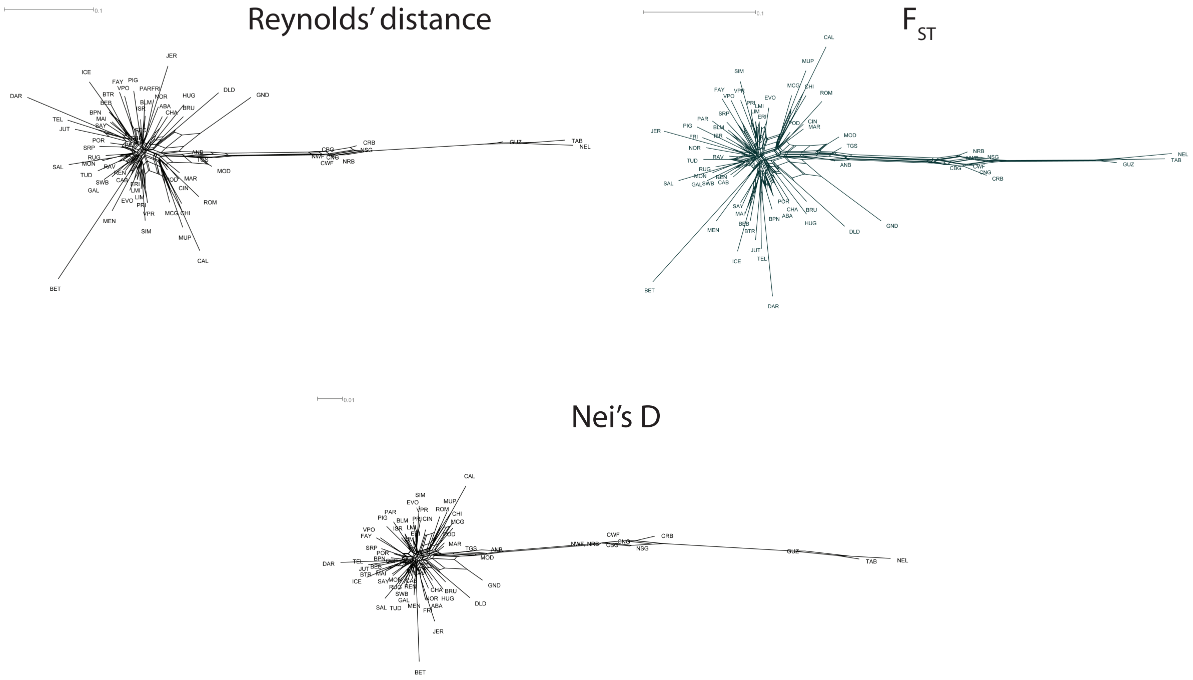
Reynolds' distance
0.1
F_{ST}
0.1
Nei's D
0.01
